# Sublethal heat reduces overall reproductive investment and male allocation in a simultaneously hermaphroditic snail species

**DOI:** 10.1098/rsos.231287

**Published:** 2024-02-07

**Authors:** Shanna M. van Dijk, Z. Valentina Zizzari, Joris M. Koene, Yumi Nakadera

**Affiliations:** Ecology and Evolution, Amsterdam Institute for Life and Environment (A-LIFE), Faculty of Science, Vrije Universiteit Amsterdam, Amsterdam, Noord-Holland, The Netherlands

**Keywords:** sex specificity, global warming, fertility, sex allocation, mating behaviour

## Abstract

The exposure to sublethally high temperature reduces reproductive performance in diverse organisms. Although this effect has been particularly emphasized for males or male reproductive functioning, it remains largely unknown whether the effect of heat on fertility is sex-specific. Here we examined the impact of sublethally high temperature on male and female functions in a simultaneously hermaphroditic snail species, *Lymnaea stagnalis*. Examining hermaphrodites is useful to evaluate the sex-specific impacts of heat exposure, since they possess male and female functions within a single individual, sharing genetic and environmental factors. Moreover, previously developed sex allocation theory allows us to compare the differential performance of sex functions. In this study, we exposed snails to 20°C (control), 24°C and 28°C for 14 days and assessed their egg and sperm production, sperm transfer, mating behaviour and growth. Both types of gamete production were significantly reduced by higher temperature, leading to an overall reduction of reproductive investment. By quantifying sex allocation, we furthermore revealed that the heat-stressed snails reduced the relative investment in their male function. This study illustrates that examining simultaneous hermaphrodites can provide significant insights for the impact of heat, and the proximate mechanism, on reproduction in diverse organisms.

## 1. Introduction

Evaluating the effects of elevated temperature on wildlife is an urgent task. It has been well established that not only the temperature at which an organism dies, i.e. critical thermal limit (CTL), but also that the temperature at which fertility is lost, i.e. thermal fertility limit (TFL), is crucial to determine the long-term viability of natural populations that experience increasing environmental temperatures (reviewed in [1]). For instance, Parrat *et al*. [2] demonstrated that, across 43 *Drosophila* species, TFLs are commonly lower than CTLs, and the estimates of TFL greatly improve the model prediction on species distribution. It implies that we would underestimate the impacts of global warming on natural populations, if we only use CTLs. Thus, studying TFLs would increase the resolution of assessments for the impact of climate changes on future biodiversity. Given the significance of TFLs, the reduced fertility under influence of heat has been reported in diverse plants and animals (e.g. [3–9]) including humans (e.g. [10]). To date, however, it remains controversial which sex is more vulnerable to increasing temperatures [11,12]. Based on several observations and hypotheses (e.g. [4,8]), male reproductive functions are often the primary target to estimate TFLs. For example, although it is not fully supported, it is a widely known hypothesis that descended testicles in the scrotum in mammals is a thermal adaptation for spermatogenesis (e.g. [13,14]). However, the assumption that male reproduction is affected more severely by heat remains to be examined. That is because females also show reduced fertility under sublethal heat exposure (e.g. [5,15,16]). Although the sex-specificity of reduced fertility under heat would significantly improve the understanding of the physiological mechanisms causing such reduction, as well as their evolutionary consequences in wildlife under climate change, the experimental insights are limited and need urgent expansion [5,17,18].

Contrasting with separate-sexed species, simultaneous hermaphrodites (hereafter hermaphrodites) represent an optimal system to test which sex is more vulnerable to heat exposure. Since these organisms possess functional male and female reproductive systems within a single body, we can determine the effects of heat on both sex functions within the same individuals. Hence, in contrast to separate-sexed species, hermaphrodites allow us to compare the sex-specific effects of heat on reproduction without cofounding effects, such as the influence of differential hormonal and genetic components, or sex-limited expression (e.g. [19]). In addition to this logistical advantage, the well-established theoretical framework to study and quantify sex allocation in hermaphrodites—how reproductive resources are allocated to the male or female function (e.g. [20–22])—offers predictive power. We hypothesize that, if the male function is more vulnerable to heat, sex allocation is expected to be female-biased compared to sex allocation of control individuals. Obviously, the backbone of sex allocation theory is selection and adaptation—the theoretical framework aims to understand why organisms change sex allocation under certain social or environmental conditions, as the consequence of selection over evolutionary time. However, we consider that the measure of sex allocation is useful to evaluate the impact of heat on reproduction in hermaphrodites, with the significant caveat that any observed changes in sex allocation under the influence of temperature are not necessarily adaptive.

To the best of our knowledge, however, there are barely any studies that examined the impact of heat on male and female functions in hermaphrodites, asking, for example, which sex is more vulnerable to heat, or if sex allocation changes depending on temperature. For example, in a self-fertilizing hermaphroditic fish, *Kryptolebias marmoratus*, Park *et al*. [18] showed that fish exposed to high temperature had a relatively smaller gonadosomatic ratio, but not testis area. Further investigation revealed the disruption of hepatic vitellogenin synthesis at high temperature, which led them to conclude that high temperature affects ovarian development more than testicular development. In a sequentially hermaphroditic self-fertilizing nematode, *Caenorhabditis briggsae*, the results indicated that the decline in fertility under higher temperature is mostly due to the compromised fertility of developing sperm, but not oogenesis or sperm count [17]. Lastly, in the hermaphroditic snail *Bulinus truncatus*, the exposure to high temperature during development tends to produce snails without a functional penis [23]. Even though such aphallic individuals produce sperm and self-fertilize, they cannot transfer ejaculate to partners. Despite the lack of male mating ability, aphallic snails were found to be as fecund as euphallic individuals and no difference in sex allocation was observed (e.g. [24,25]). Although the number of studies is fairly limited and all the study species were self-fertilizing hermaphrodites (indicating their male investment is low, regardless of temperature), previous studies indeed suggest that examining hermaphrodites could tell which sex function is more affected by heat, motivating us to examine the impacts of elevated temperature on hermaphrodites and their sex allocation.

We examined the impacts of heat exposure on reproduction in a hermaphroditic snail species, *Lymnaea stagnalis*. It has been well established in this species that exposure to higher temperatures alters egg production as well as immune response [26–32], memory formation [33,34], food consumption [35], synaptic transmission [36], and respiration [37]. Also, the reproductive biology of *L. stagnalis* is well studied and their reproductive performance is readily quantifiable (e.g. sperm count [38], fecundity [39], male and female mating behaviour [40,41]. In addition, this species prefers using sperm from mating partners for fertilization (i.e. outcrossing), and is capable of self-fertilization when a mate is not available or when it runs out of sperm from partners [42–44]. Previous studies showed that these snails alter sex allocation depending on mating history, but not mating frequency [45,46]. In this study, we focused on evaluating the difference between male and female functions, by comparing egg and sperm production by the same individuals. In addition, we measured the effect of heat on somatic growth and mating behaviour. We report here that the snails exposed to 28°C for two weeks reduced their overall investment in reproduction and their male function was diminished more than their female function. In addition, we revealed that their drastically reduced sperm production did not discourage them from mating in the male role.

## 2. Material and methods

We used adult *L. stagnalis* from the long-standing laboratory culture at Vrije Universiteit Amsterdam. During rearing, these snails were kept in flow-through tanks with aerated low-copper water at 20 ± 1°C and a light : dark cycle of 12 : 12 h. They were fed with broadleaf lettuce and fish flakes (Tetraphyll, TetraGmbH) ad libitum*.* We used an age-synchronized cohort of snails, which was three months old at the start of the experiment and fully sexually matured as evidenced by their egg laying capability. This species is simultaneously hermaphroditic and shows unilateral mating, meaning that, in a single mating event, one snail acts as male (sperm donor) and its partner as female (sperm recipient). When motivated, they can swap their sex roles immediately after each mating [45].

### 2.1. Heat exposure setup

**Figure 1.**
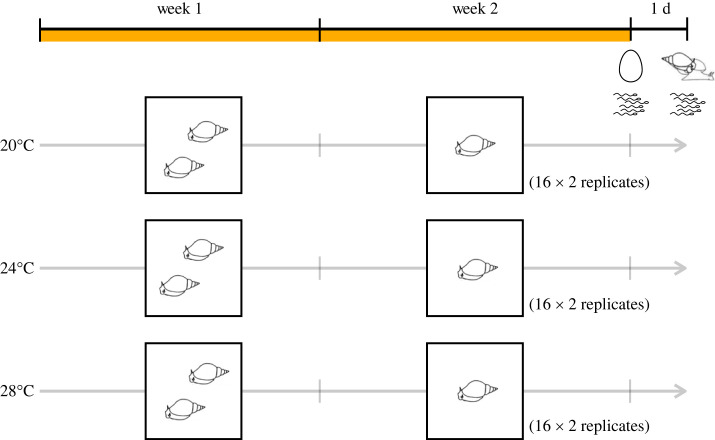
Experimental design. In Week 1, we allowed the snails to mate in pairs, so that they used up gametes produced and stored prior to heat treatment. In Week 2, we removed one snail from each container to keep the focal snail isolated. Half of the focal snails were used to measure the production of both types of gametes, as indicated by the pictograms of an egg and sperm, at the end of Week 2. On the following day, we let the other half of the focal snails copulate with the laboratory snails to measure mating behaviour and sperm transfer (see mating snail and sperm pictogram). We used two aquariums with eight perforated containers for each temperature treatment, and conducted the same experiment twice to obtain sufficient sample size.

We randomly allocated 48 snails to 20°C, 24°C and 28°C for 14 days (16 snails per temperature treatment; figure 1). We set 20°C as control, because this is the standard rearing temperature and near their thermal optimum [40,47,48]. We chose 24°C and 28°C as simulated warm and extremely warm summer conditions in shallow water bodies (see also [28]). Based on the previous studies showing that this species can survive from 4°C to 40.5°C (e.g. [49,50]), we consider the range of temperature we chose as sublethal. We chose to examine the effect of continuous heating for 14 days, rather than a brief heat shock, as water is slow to increase or decrease its temperature compared to air. Moreover, this duration is long enough to evaluate the effect of heat on sperm production, since their spermatogenesis takes less than 10 days [51,52]. To achieve these heat treatments, we used six aquariums (*ca* 15 l) with heaters. These aquariums have a slow flow-through of aerated water, similar to the standard breeding tanks. Within an aquarium, we placed 8 perforated containers (400 ml) to monitor the snails. To acclimate the snails to a designated temperature, we put snails in a closed container with water at 20°C, and placed the container in the aquarium for a few hours until the water inside the container had the same temperature as the surrounding aquarium water. Then, we exchanged the closed containers with perforated containers to initiate heat exposure. Throughout the experiment, we fed the snails with a lettuce disc (*ca* 19.6 cm^2^) per day *per capita*. Due to the limited number of available aquariums with individual thermostats, we ran the same experiment twice to ensure a high enough sample size (*N* per treatment = 32, total *N* = 96).

In the first week of heat exposure, we kept two snails together in a container, allowing for depletion of sperm that they had produced and stored in their seminal vesicles before the exposure. We randomly assigned one snail in a pair as focal, by placing a small amount of nail polish on the non-focal individual. Then, at the start of the second week, we removed the non-focal snails from the containers. After exposing the focal snails to the designated temperature for a total of 14 days (see figure 1), we quantified their sperm and egg productions at the end of Week 2. Furthermore, on Day 15, we provided a mating partner to each focal snail, in order to examine their mating behaviour as well as sperm transfer. In addition, we measured growth of all the focal snails. Based on the sperm and egg production data, we also compared the total investment in reproduction and determined sex allocation across treatments. We describe how we collected these data in the following.

### 2.2. Growth

Before the start of the temperature treatment, we measured the shell length of focal snails as proxy of body size [53], using Vernier callipers (min: 0.01 mm). At the end of the exposure, we measured the shell length of focals again to see how much the snails had grown in two weeks.

### 2.3. Egg production

At the start of Week 2, we provided new containers to each focal individual. At the end of the temperature exposure, we randomly assigned half of the focal snails for measuring egg and sperm production. From the selected focal individuals, we collected all the egg masses laid in Week 2. We scanned the collected egg masses using a flatbed scanner and glass plates with spacers (Canon LiDE 220; see more details in the video instruction in [39]). The scanned images of egg masses were used to count the number of egg masses and number of eggs laid using ImageJ (ver. 1.53t) [54]. Since this species typically lays 1–3 egg masses per week, and each egg mass contains usually 50–120 eggs, we counted the number of eggs in each egg mass based on the scanned images, then calculated the total number of eggs laid by each individual. In addition, we calculated the average number of eggs per egg mass by dividing the total number of eggs by the number of egg masses. Contrasting to the number of egg masses and the total number of eggs, the average number of eggs per egg mass is not exactly fecundity, rather an egg mass property. That is, we cannot distinguish whether the snails show a reduced number of eggs to make an egg mass, or they alter the number of eggs per egg mass. Therefore, we consider the analysis of average number of eggs per egg mass as supplemental.

### 2.4. Sperm production

The focal snails that were used for quantifying egg production, immediately after the treatment, were subsequently used to dissect out their seminal vesicles in order to estimate how many sperm they produced and stored in Week 2. We used the method of sperm counting published, with a few modifications [38]. First, we euthanized the snails by slowly injecting *ca* 2 ml of 50 mM MgCl_2_ through their foot into the haemocoel using a syringe and needle (30G×½″). Next, using a coarse forceps, we removed the shell and pinned the soft body onto a dissection plate. Then, using a fine forceps and scissors, we carefully dissected out the whole seminal vesicles and placed these into 800 µl of *Lymnaea* saline solution in a 2 ml tube. Within the solution, we tore apart the duct with a fine forceps and then vortexed for 30 s. Next, we transferred the duct to a new tube with 400 µl of saline and vortexed it for 30 s again. We repeated this last step one more time, removed the duct and collected all the solutions into the first tube. After vortexing it for another 30 s, we took 5 µl of sperm suspension to count the sperm heads, using a Neubauer improved cell counter, and repeated this counting four times for each sample. Lastly, we applied the formula in Loose & Koene [38] to estimate the number of sperm in the original sperm suspension (depth: 0.1 mm, the number of squares counted: 5, the area of each square: 0.04 mm^2^).

### 2.5. Mating behaviour

One day after Week 2, we let the remaining set of focal snails copulate with partner snails to measure mating behaviour and sperm transfer. At the end of the temperature treatment, we kept these snails in a flow-through tank at 20°C for one day. We also isolated partner snails for four days in perforated containers placed in the same flow-through tank. For identification purposes, we put a small amount of nail polish on the shell of partners. Since the focal snails were isolated for eight days, they were fully motivated to copulate as male [55,56], and more so than their four-day isolated partners.

On the day of mating observation, we placed one focal and one partner snail together in a container filled with *ca* 400 ml of water. The mating observation was conducted for six hours (09.00–15.00) at the control temperature (20 ± 1°C). According to the series of stereotypical mating behaviours in this species [57], we checked all the pairs every 10 min and scored whether (i) they were not in contact, (ii) the focal (or partner) was crawling on the partner's shell (mounting), (iii) the focal was probing or inseminating. Since insemination usually takes 20–60 min, this sampling interval ensured not missing any copulation.

Based on the mating behaviour data, we counted how many focal individuals mated, and which sex roles they performed first (male or female). We also calculated the duration of mating latency (how long they took to initiate courtship behaviour), that of courtship (from the start of contact until the start of insemination), and that of insemination. For the analyses of mating behaviour, we only used the cases where focal snails acted as male first. When a focal snail acts as female first, then swaps its sex role to inseminate the partner in its second mating, it is difficult to define when the focal snail started courtship behaviour, as they are often in contact already (fig. 3 in [45]). Moreover, mating as female first has been shown to affect sperm transfer [43,44].

### 2.6. Sperm transfer

Immediately after the focal snail inseminated their partner, we took out the partner and dissected out the vaginal duct which was extensively swollen with ejaculate received (see the method above). We placed the extracted duct in the tube with 400 µl of saline and tore it apart to release the sperm transferred. We followed the same protocol of sperm counting as above, except that the total amount of sperm suspension was 1200 µl, not 1600 µl. That is because the number of sperm transferred was expected to be less than that produced, as supported by our data in this study.

### 2.7. Statistics

We carried out all the statistical analyses in R (ver. 4.2.1) [58]. Throughout the experiment, we collected the data for growth, egg production (the number of egg masses, the total number of eggs, the average number of eggs per egg mass), sperm production, sperm transfer and mating behaviour (mating rate, mating role, mating latency, courtship duration, insemination duration). To test if there is any difference between treatments, we used statistical models with Treatment (20, 24 and 28°C) and Run (Run 1 and 2) as fixed factors including interaction. To compare the growth between treatments, we calculated the shell length differences before and after exposure and ran a GLM with Gaussian distribution. To explain the difference of body size we observed between experimental runs, we also ran the same test for the shell length at the start of experiments. To compare the egg production, we used a GLM with Poisson distribution for the number of egg masses, and a GLM with Gaussian distribution for the number of eggs and the number of eggs per egg mass. For the number of egg masses, we chose Poisson distribution, since this is a discrete variable. We also assessed overdispersion by calculating the ratio of residual deviance to degree of freedom, which was 0.264, indicating a relatively low level of overdispersion. When there was a significant difference, we applied a Tukey *post hoc* test. To test the sperm production and sperm transfer, we used GLMs with Gaussian distribution and Tukey *post hoc* tests. For mating rate and mating role, we used chi-square tests. Lastly, for the other mating behaviour data, we applied Kruskal–Wallis tests (without including Run as fixed factor), since these variables were not normally distributed.

The change in sperm and egg production in response to temperature further motivated us to examine the change in total reproductive investment as well as sex allocation. To do so, we first made the number of eggs produced and sperm production data comparable by standardizing them (mean 0, SD 1) and adding 3 to all the values to make them non-negative. Then, we consider the sum of standardized egg and sperm production as total reproductive investment. For sex allocation, we used the ratio of standardized sperm production divided by total reproductive investment. For these two variables, we again applied GLMs with Gaussian distribution and Tukey *post hoc* tests.

## 3. Results

Throughout the experiments, two out of 96 snails died in the 28°C treatment of Run 2. These two snails were excluded from all the analyses. See the sample sizes of each measurement in the figure legends.

### 3.1. Growth

**Figure 2.**
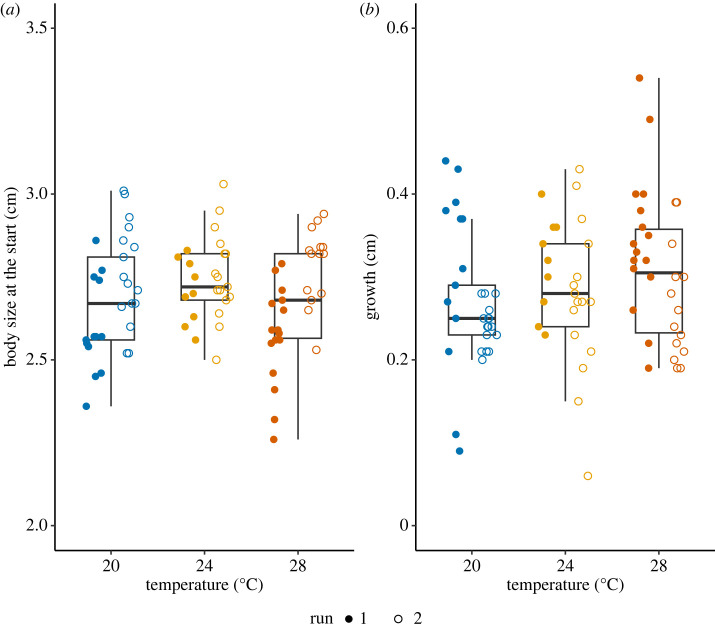
Growth across heat treatments. (*a*) The body size at the start of experiment. The individuals in Run 2 were larger than those in Run 1, since they were 2 weeks older. (*b*) Growth. We plot the difference of shell length at the start and end of the experiment as growth. The box plots show median, first and third quartiles and range of data points (20°C: *N*_Run1_ = 13, *N*_Run2_ = 16; 24°C: *N*_Run1_ = 9, *N*_Run2_ = 16; 28°C: *N*_Run1_ = 16, *N*_Run2_ = 14).

Due to handling errors, the sample sizes for growth data were 29 for 20°C, 25 for 24°C, and 30 for 28°C. Within the duration of two weeks, we did not detect any difference in growth between treatments (GLM, *F*_2,81_ = 1.88, *p* = 0.159, figure 2), although there was a significant difference between Run (GLM, *F*_1,80_ = 11.91, *p* = 0.001, Interaction: *F*_2,78_ = 0.30, *p* = 0.739, figure 2). During the course of two weeks, the snails gained 9.63% of body size on average. Note that, since we used the same age cohort of snails for both runs, they had a two-week age difference, and the size of snails in Run 2 was indeed larger at the start of the experiment (GLM, Run: *F*_1,80_ = 24.83, *p* < 0.001, figure 2).

### 3.2. Egg production

**Figure 3.**
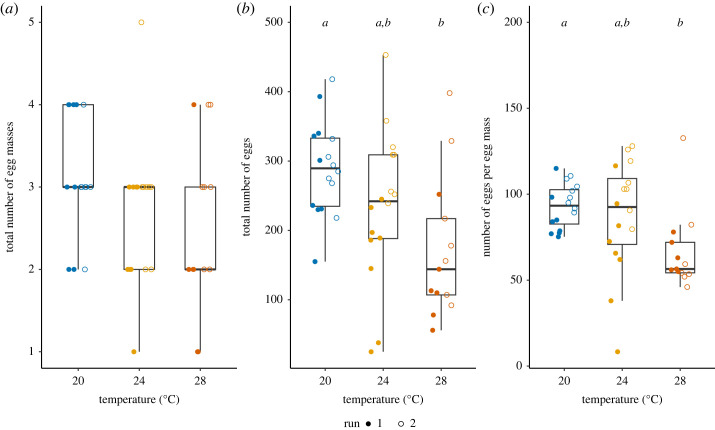
Egg production across heat treatment. (*a*) Total number of egg masses laid in Week 2. (*b*) Total number of eggs laid in Week 2. (*c*) The average number of eggs per egg mass. The letters above box plots indicate the outcome of Tukey *post hoc* tests (total number of eggs: 20°C versus 24°C, *p* = 0.236, 20°C versus 28°C, *p* = 0.002, 24°C versus 28°C, *p* = 0.171; average number of eggs per egg mass: 20°C versus 24°C, *p* = 0.766, 20°C versus 28°C, *p* = 0.008, 24°C versus 28°C, *p* = 0.053). The box plots show median, first and third quartiles and range of data points (20°C: *N*_Run1_ = 8, *N*_Run2_ = 8; 24°C: *N*_Run1_ = 8, *N*_Run2_ = 8; 28°C: *N*_Run1_ = 6, *N*_Run2_ = 7).

Two snails in the 28°C treatment did not lay eggs. Therefore, we did not include them to compare the egg production across treatments. There was no difference in the number of egg masses laid across treatments (GLM, Treatment: *p* = 0.612, Run: $\chi_{1}^{2}=0.71$, *p* = 0.400, Interaction: $\chi_{2}^{2}=1.24$, *p* = 0.537, figure 3*a*), but the total number of eggs laid was significantly lower in the snails in 28°C compared to those in 20°C (GLM, Treatment: *F*_2,42_ = 7.65, *p* = 0.002, Run: *F*_1, 41_ = 13.36, *p* = 0.001, Interaction: *F*_2,39_ = 2.75, *p* = 0.076, figure 3*b*). This was also reflected in the number of eggs per egg mass, which showed the same pattern as the total number of eggs laid (GLM, Treatment: *F*_2,42_ = 6.23, *p* = 0.004, Run: *F*_1,41_ = 10.45, *p* = 0.002, Interaction: *F*_2,39_ = 2.68, *p* = 0.081, figure 3*c*). Even though not statistically significant, we highlight the difference between runs at 24°C. These two groups of snails had an age difference of two weeks (figure 3), and this becomes more prominent in our sex allocation analysis below. Compared to the control (20°C), the number of eggs produced was reduced by 40.6% on average at 28°C.

### 3.3. Sperm production

**Figure 4.**
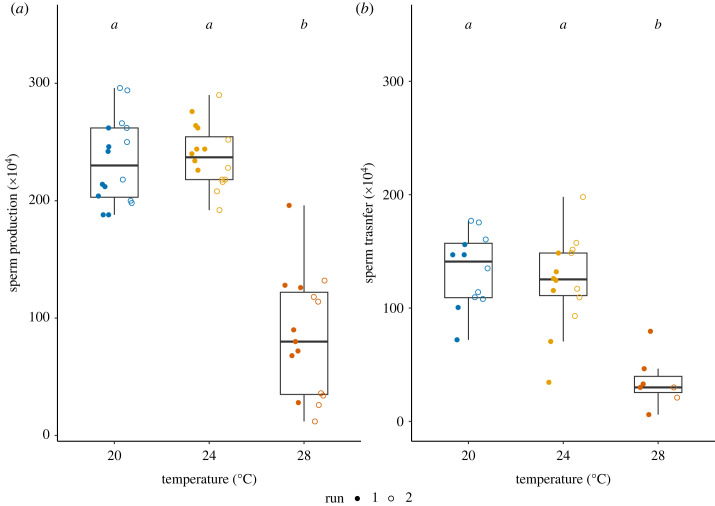
Sperm production across heat treatment. (*a*) The estimated number of sperm produced in Week 2. (*b*) The estimated number of sperm transferred to a mating partner on Week 2 + 1d. The letters above bar plots indicate the outcome of Tukey *post hoc* tests (sperm production: 20°C versus 24°C, *p* = 0.943, 20°C versus 28°C, *p* < 0.001, 24°C versus 28°C, *p* < 0.001; sperm transfer: 20°C versus 24°C, *p* = 0.733, 20°C versus 28°C, *p* < 0.001, 24°C versus 28°C, *p* < 0.001). The box plots show median, first and third quartiles and range of data points (sperm production, 20°C: *N*_Run1_ = 8, *N*_Run2_ = 8; 24°C: *N*_Run1_ = 8, *N*_Run2_ = 8; 28°C: *N*_Run1_ = 8, *N*_Run2_ = 7; sperm transfer, 20°C: *N*_Run1_ = 5, *N*_Run2_ = 7; 24°C: *N*_Run1_ = 7, *N*_Run2_ = 7; 28°C: *N*_Run1_ = 5, *N*_Run2_ = 2).

We detected a significant reduction of sperm production in the snails at 28°C (GLM, Treatment: *F*_2,44_ = 82.91, *p* < 0.001, Run: *F*_1,43_ = 0.44, *p* = 0.510, Interaction: *F*_2,41_ = 2.80, *p* = 0.072, figure 4*a*). On average, compared to the control, the snails at 28°C produced 64.1% less sperm.

### 3.4. Sperm transfer

Because we can measure sperm transfer only when the focal snails had copulated, the sample size is smaller and varying (see the caption of figure 4). The data show a very similar pattern to the sperm production results: the snails at 28°C transferred significantly less sperm to their mating partner (Treatment: *F*_2,30_ = 20.88, *p* < 0.001, Run: *F*_1,29_ = 2.08, *p* = 0.160, Interaction: *F*_2,27_ = 0.91, *p* = 0.413, figure 4*b*). On average, compared to the control, the snails at 28°C transferred 73.7% less sperm.

### 3.5. Mating behaviour

**Figure 5.**
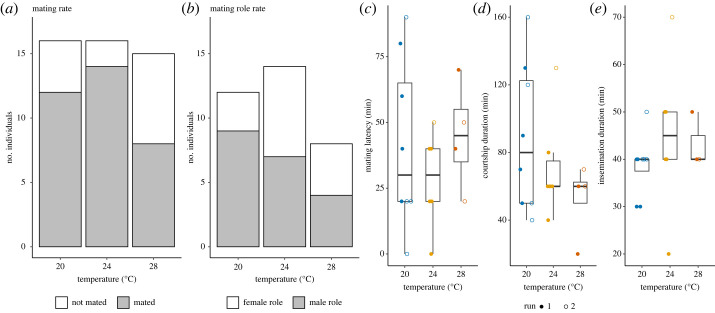
Mating behaviours of heat-treatment snails mating with control partners. (*a*) Mating rate after heat treatment. We counted how many focal individuals mated with control, non-heat-treated snails. (*b*) Mating role rate after heat treatment. We counted how many focal snails acted as male or female in their first mating with control snails. (*c*) Mating latency. We plotted the duration from the start of the mating trial until a focal snail initiated male courtship behaviour. (*d*) Courtship duration. We measured the duration of courtship from mounting to the end of probing. (*e*) Insemination duration. The *y*-axis indicates the duration of ejaculate transfer. The box plots show median, first and third quartiles and range of data points (20°C: *N*_Run1_ = 4, *N*_Run2_ = 4; 24°C: *N*_Run1_ = 5, *N*_Run2_ = 1; 28°C: *N*_Run1_ = 2, *N*_Run2_ = 2).

The mating rate did not differ between treatments, when they mated with non-heat-treated, control partners (Treatment: $\chi_{2}^{2}=4.60$, *p* = 0.100, figure 5*a*). Their mating roles also did not differ significantly between treatments (Treatment: $\chi_{2}^{2}=2.00$, *p* = 0.367, figure 5*b*). For the remaining mating behaviour data, we had to exclude one sample (Run 2, 28°C) that we prematurely interrupted for sperm counting; the focal snail had not transferred an ejaculate yet. Nonetheless, we did not find any significant difference in mating latency (Kruskal–Wallis test, $\chi_{2}^{2}=1.16$, *p* = 0.559, figure 5*c*), courtship duration (Kruskal–Wallis test, $\chi_{2}^{2}=1.85$, *p* = 0.397, figure 5*d*) and insemination duration (Kruskal–Wallis test, $\chi_{2}^{2}=1.73$, *p* = 0.420, figure 5*e*).

### 3.6. Investment for reproduction and sex allocation

**Figure 6.**
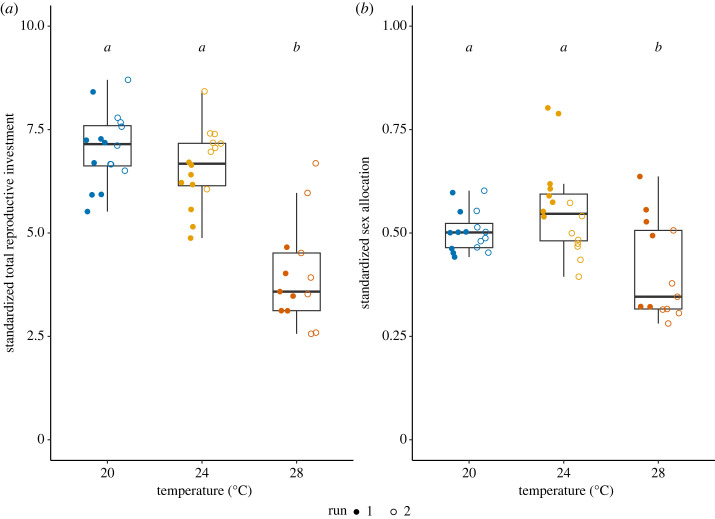
Investment for reproduction and sex allocation. (*a*) Investment for reproduction is based on the number of eggs and sperm produced. Since we standardized the measurements to sum them up, these values are comparable across treatments, but arbitrary (see §2). (*b*) Sex allocation is the ratio of sperm production divided by investment for reproduction. Higher values on the *y* axis indicate male biased sex allocation, and lower values female biased sex allocation. The letters above bar plots indicate the outcome of Tukey *post hoc* tests (investment for reproduction: 20°C versus 24°C, *p* = 0.383, 20°C versus 28°C, *p* < 0.001, 24°C versus 28°C, *p* < 0.001; sex allocation: 20°C versus 24°C, *p* = 0.247, 20°C versus 28°C, *p* = 0.021, 24°C versus 28°C, *p* < 0.001). The box plots show median, first and third quartiles and range of data points (20°C: *N*_Run1_ = 8, *N*_Run2_ = 8; 24°C: *N*_Run1_ = 8, *N*_Run2_ = 8; 28°C: *N*_Run1_ = 6, *N*_Run2_ = 7).

There was a significant reduction in total reproductive investment in the 28°C treatment (Treatment*: F*_2,42_ = 44.51, *p* < 0.001, Run: *F*_1,41_ = 8.61, *p* = 0.006, Interaction: *F*_2,39_ = 0.66, *p* = 0.521, figure 6*a*). Sex allocation was also different across treatments and as well as Run and Interaction (Treatment: *F*_2,42_ = 12.82, *p* < 0.001, Run: *F*_1,41_ = 13.54, *p* = 0.001, Interaction: *F*_2,39_ = 4.35, *p* = 0.027, figure 6*b*). The male allocation in the snails exposed to 28°C was significantly reduced, compared to the control treatment. The interaction was most likely due to the variation between runs in the 24°C treatment (figure 6; electronic supplementary material, table S1), implying that this may be an effect of age.

## 4. Discussion

We found that exposing snails to sublethal temperature significantly reduced both egg and sperm productions, and that their male function was more vulnerable than their female function. Also, we found that, despite of their reduced sperm production and transfer, their male mating motivation was not affected. Lastly, we did not observe any effect of heat on growth. In sum, heat sensitivity of male fertility corresponds to previous studies in separate-sexed species, but our study also highlights the importance of evaluating female fertility under heat, since female function was indeed affected by heat in this species.

We found that, compared to the female function, the male function of *L. stagnalis* is more sensitive to heat exposure. The control group invested equally in male and female reproduction, which is in accordance with previous studies looking at sex allocation in this species [46,59]. We found that the total investment in reproduction especially declined in the snails exposed to 28°C. This pattern supports the consensus that reproduction is vulnerable to sublethal heat exposure [1,12]. Our results also indicate that snails exposed to 28°C allocated proportionally more to their female function, compared to the control snails (figure 6*b*). The shifted sex allocation and diminished sperm production and transfer lead us to conclude that the male function is more vulnerable to heat stress in this species. Again, this observation matches with other studies in separate-sexed species (e.g. [8]; reviewed in [1,12]). We designed the experiment with the aim to measure the consequence of heat on spermatogenesis, even though this species continuously produces sperm after maturation. Previous work has shown that one week of isolation suffices to fully replenish the components of ejaculate in this species, increasing their male mating motivation [55,56]. Thus, we are confident that snails in 28°C treatment did not fully replenish their sperm reserves. Clearly, it remains to be determined which spermatogenesis stage is affected and whether the produced sperm are viable.

Our study highlights the importance of measuring fertility of both sexes under heat. As Iossa [11] pointed out, the pathway responsible for heat sensitivity of fertility is likely to be sex-specific, and further investigation of both sex functions is needed [12]. We indeed detected that egg production was also decreased under heat (figure 3; also see [28,30]). This reduction probably occurs because the heat-exposed snails deposited fewer eggs in an egg mass, rather than changing their egg laying frequency (figure 3*c*). Similar to sperm production, we did not measure the quality of eggs produced under heat, and do not know which stage of egg production was affected by heat. Since *L. stagnalis* can store and use sperm from mating partners for approximately three months [42,44], the snails should have had plenty of sperm to fertilize during the treatment, although we cannot exclude the possibility that the stored sperm deteriorated under heat. In addition, we did not find an effect of heat on growth in our experiment, although the exposure to sublethally high temperature would facilitate the growth of this species, due to their increased metabolic rate. Based on the previous studies (e.g. [28,30,50]), it is likely that, if we had kept the snails under heat treatment for a longer period or larger temperature differences, we would have detected such a difference. The trend that the snails at 28°C show might be seen as a confirmation for this previously reported response. Even though there is a positive correlation between body size and female fecundity in this species [46], we observed a significant reduction in egg production under heat, showing the impact of heat on female fertility (figure 3).

In addition, the observed interaction with heat treatment and experimental runs in sex allocation might imply that the temperature vulnerability is age specific (figure 6*b*; electronic supplementary material, table S1), although further investigation is absolutely required to test this hypothesis. The two-week age difference was a logistic limitation, rather than an intended treatment. To quantify the shift of sex allocation depending on temperature and age, we would need a wider age range and higher sample sizes for each treatment. Because of age-depending sex allocation [40,60] and performance (e.g. memory formation [61]) in this species, such age-dependent responses would be plausible and a fruitful follow-up to expand the understanding for the impacts and physiological mechanism of heat exposure on male and female fertility [62].

We found that sperm production was reduced under heat and this reduction strongly influenced the sperm transfer of *L. stagnalis* (figure 4), but not their mating motivation and behaviour (figure 5). Since this species transfers sperm at the end of insemination [63], it is expected that the insemination duration is not correlated with the number of sperm transferred. Also, we emphasize that the snails at 20°C and 24°C transferred approximately 50% of sperm stored, and the snails at 28°C used almost all sperm they had (figure 4). Moreover, such unaffected male mating motivation in *L. stagnalis* was also observed in a different context. When snails receive seminal fluid proteins, they significantly reduce the number of sperm transferred in a subsequent mating [43,44], although their male mating motivation stays unchanged [40]. The observed mismatch of reduced sperm production and unchanged male mating motivation in this study is particularly concerning for field populations. Although male mating could increase the fitness of an individual, for heat-stressed snails mating would entail higher costs and risks. A previous study in this species showed that male mating is as costly as female mating [59]. The energetic cost for male mating is not only sperm [64]. For example, the male acting individuals also have to convince the partner to copulate and perform courtship behaviours, which usually take 1–4 h under laboratory conditions. Previous studies indeed show that the female acting snails show mating avoidance behaviours; thus, they are not always passive and accepting (e.g. [41,65]). Furthermore, the heat-stressed snails could not mate as often as the control ones in future, since their sperm reserve is empty and replenishment would be slower. Lastly, mating in the field is always associated with predation and infection risks. That is especially relevant, as other studies show that heat exposure compromises their immune defences (e.g. [30,32]). Therefore, although it might still be beneficial for the snails to mate even if exposed to heat stress, a mismatch of sperm production and mating motivation could have several negative consequences, outweighing the benefits. It is worth noticing that we chose to let the snails have a day at 20°C before mating, to test the effect of reduced sperm production on mating behaviour. This ‘rest’ day might have affected their male mating motivation and behaviour. For future research, it would be interesting to directly examine how heat affects mating behaviour.

This study demonstrated that examining hermaphrodites provides unique and vital insights on the sex differences under heat, which can facilitate further investigations beyond the boundary of reproductive modes. As commonly expected or assumed in separate-sexed species [1,4,11,12], the male function of *L. stagnalis* is more sensitive to elevated temperature, which means that the proxy mechanism of those responses can be shared across a wide range of species. As for many hermaphroditic snails and slugs, *L. stagnalis* produces sperm and eggs in the same organ called ovotestis [46,66]. Although the ovotestis is a particularly interesting organ, our current understanding of how this organ functions in gastropods is limited, in terms of gene expression, distribution of oo- and spermatogenesis sites, or the fate determination of germ cells. This study paves the path to investigate the proximate mechanisms of reduced male and female fertility under heat in a hermaphroditic species and to predict the implications in natural populations. Moreover, with temperature projected to increase in future, we hope this study motivates further studies investigating the impact of heat exposure in a wide range of hermaphrodites.

## Data Availability

The data and R code of this research are openly available (https://doi.org/10.48338/VU01-IZJOOF) [67]. Supplementary material is available online [68].
